# Partitioning and recovery of proteases from lizardfish (*Saurida micropectoralis*) stomach using combined phase partitioning systems and their storage stability

**DOI:** 10.1039/d3ra01069d

**Published:** 2023-05-15

**Authors:** Sakonwat Kuepethkaew, Sappasith Klomklao, Yi Zhang, Benjamin K. Simpson

**Affiliations:** a Biotechnology Program, Faculty of Agro and Bio Industry, Thaksin University Phatthalung Campus Pa-Phayom Phatthalung 93210 Thailand; b Department of Food Science and Technology, Faculty of Agro and Bio Industry, Thaksin University Phatthalung Campus Pa-Phayom Phatthalung 93210 Thailand sappasith@tsu.ac.th +66 7460 9618 +66 7460 9618; c Department of Food Science, The Pennsylvania State University University Park PA 16802 USA; d Department of Food Science & Agricultural Chemistry, McGill University 21111 Lakeshore Road Ste-Anne-de-Bellevue QC H9X 3V9 Canada

## Abstract

Partitioning and recovery of proteases from stomach extract (SE) and acidified stomach extract (ASE) of lizardfish using a three-phase partitioning (TPP) system in combination with an aqueous two-phase system (ATPS) was optimized. The highest yield and purity were obtained in the interphase of the TPP system, which consisted of a SE or ASE to *t*-butanol ratio of 1.0 : 0.5 in the presence of 40% (w/w) (NH_4_)_2_SO_4_. Both TPP fractions were further subjected to ATPS. Phase compositions of ATPS including PEG molecular mass and concentrations as well as types and concentrations of salts influenced protein partitioning. The best ATPS conditions for protease partitioning into the top phase from TPP fractions of SE and ASE were 15% Na_3_C_6_H_5_O_7_–20% PEG1000 and 20% Na_3_C_6_H_5_O_7_–15% PEG1000, which increased the purity by 4-fold and 5-fold with the recovered activity of 82% and 77%, respectively. ATPS fractions of SE and ASE were subsequently mixed with several PEGs and salts for back extraction (BE). BE using 25% PEG8000–5% Na_3_C_6_H_5_O_7_ gave the highest PF and yield for both ATPS fractions. SDS-PAGE investigation revealed that the decrease in contaminating protein bands was observed after the combined partitioning systems. BE fractions of SE and ASE were quite stable at −20 and 0 °C up to 14 days. Therefore, the combination of TPP, ATPS and BE could be effectively applied to recover and purify proteases from the stomach of lizardfish.

## Abstract 2

Partitioning and recovery of proteases from stomach extract (SE) and acidified stomach extract (ASE) of lizardfish using a three-phase partitioning (TPP) system in combination with an aqueous two-phase system (ATPS) was optimized.

## Graphical abstract

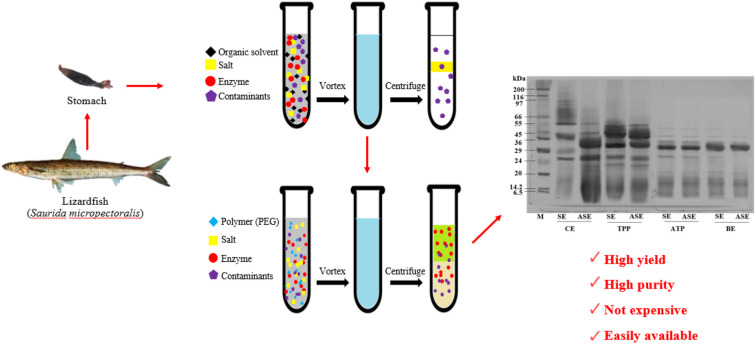

## 1. Introduction

Surimi, concentrated myofibrillar proteins obtained by successive washing of minced fish, is a useful ingredient for producing various processed foods with unique textural properties. Surimi-based products such as crab legs, chikuwa, fish balls, sausages, *etc.* have gained increasing attention because of the preferred textural properties and high nutritional value.^1^ Thailand is one of the most important surimi producing countries in Southeast Asia. Lizardfish (*Saurida* spp.) are tropical fish that have been commonly used as a potential raw material for surimi production in Thailand due to their availability, white color, good flavor, and good gel forming ability.^2^ During surimi processing, high amounts of processing by-products, especially viscera are discarded. Fish viscera is a potential natural source for recovering enzymes such as proteases that may have some unique properties for industrial applications *e.g.* in detergent, food, pharmaceutical, leather and silk industries.^3^ Stomach protease, especially pepsin can be recovered and applied mainly for hydrolysis purposes. Pepsin can be extracted and recovered from fish by-products, especially fish stomach, which constitute 5% of fish weight.^4^

Nowadays, liquid–liquid extraction like a three-phase partitioning (TPP) and aqueous two-phase system (ATPS) are promising alternatives for separating biomolecules, especially proteins and enzymes.^5,6^ Both TPP and ATPS are relative simple and inexpensive, are easily operated and scaled-up.^6^ TPP is a simple bioseparation and purification technique in which a salt (*e.g.* ammonium sulphate) and water miscible aliphatic alcohol (*e.g. t*-butanol) are added to an aqueous solution containing proteins. Under optimized conditions, three phases are formed within an hour.^6^ Biomolecules are recovered in a purified form at the interphase, while the contaminants such as lipids, phenolics and some detergents mostly partition in *t*-butanol (top phase) or aqueous phase (bottom phase).^7^ This method is scalable and can be applied directly with crude suspensions. For ATPS, it has been an attractive technique for recovery of biological materials over other methods (precipitation, column chromatography and electrophoresis) since it constitutes gentle environmental conditions containing high water content in both liquid phases up to 70–90%.^5^ ATPS is generally formed by mixing aqueous conditions of two or more incompatible polymers or of polymer and salt above critical concentration.^5,8^ ATPS can remove contaminants, such as nucleic acids and undesirable proteins. The application of TPP or ATPS in downstream processing has been focused on partitioning, separation and recovery of various enzymes including pepsin,^3^ lipase,^6^ trypsin,^8^*etc.* In general, the single system has been implemented for enzyme partitioning and recovery. The use of combined partitioning systems, *e.g.* TPP and ATPS could be an advantageous separation method to increase the purity of the target enzyme as well as to increase the recovery yield.^6,9^ However, there is no information regarding the use of combined TPP and ATPS for partitioning and recovery of proteases from lizardfish stomach. Therefore, the aim of this study was to optimize the separation process of proteases from the lizardfish stomach by using TPP in combination with ATPS. The storage stability of the partitioned enzymes with different temperatures and times was also investigated.

## 2. Materials and methods

### 2.1 Chemicals

Acetone, polyethylene glycol (PEG) 1000, PEG2000, PEG4000, PEG8000, *tert*-butanol (*t*-butanol) and tris(hydroxymethyl)aminomethane were obtained from Merck (Darmstadt, Germany). Hemoglobin from bovine blood, bovine serum albumin (BSA), wide range molecular weight markers and Coomassie Brilliant Blue G-250 were purchased from Sigma Chemical Co. (St. Louis, MO, USA). Sodium dodecyl sulphate (SDS) was obtained from Fluka (Buchs, Switzerland). *N*,*N*,*N*′,*N*′-Tetramethyl ethylene diamine (TEMED) was purchased from Bio-Rad Laboratories (Hercules, CA, USA). The salts and other chemicals with the analytical grade were procured from Merck (Darmstadt, Germany).

### 2.2 Preparation of crude extract

Lizardfish (*Saurida micropectoralis*), off-loaded approximately 24–36 h after capture from the Andaman Sea of Thailand were transported to Thaveelarp Fisheries Ltd, Part. Trang, Thailand. Upon arrival, lizardfish stomach was separated, collected and transported to the Department of Food Science and Technology, Thaksin University, Phatthalung, within 2 h. The samples were placed in ice using a sample : ice ratio of 1 : 2 (w/w). The stomach was defatted with acetone and used for protease extraction according to the method Kuepethkaew *et al.*^6^ The dry powder referred to as “defatted stomach powder” was stored at −20 °C until used.

To prepare the crude extract, the defatted stomach powder was suspended in 50 mM Tris–HCl, pH 7.0 containing 0.2% (v/v) Tween 20 at a ratio of 1 : 9 (w/v) and stirred continuously at 4 °C for 30 min.^10^ The suspension was centrifuged for 30 min at 4 °C at 5000 × *g* to remove the tissue debris. The supernatant was collected and referred to as “stomach extract, SE”. To activate stomach protease, SE was adjusted to pH 2.0 with 1.0 M HCl and the mixture was allowed to stand at 4 °C for 30 min.^3^ The suspension was centrifuged at 4 °C for 30 min at 10 000 × *g* using a refrigerated centrifuge (Sorvall Contifuge Stratos, Thermo Fisher Scientific, Waltham, MA, USA). The collected supernatant was referred to as “acidified stomach extract, ASE”.

### 2.3 Enzyme assay and protein determination

Enzyme activity was examined using hemoglobin as the substrate. The activity was tested at pH 2.0 and 40 °C for 15 min as described by Klomklao *et al.*^11^ and Kuepethkaew *et al.*^10^ The oligopeptide content in the supernatant was determined by the Lowry method^12^ using tyrosine as a standard. One unit of activity was defined as that releasing 1 μmol of tyrosine per min (μmol Tyr per min). A blank was run in the same manner, except that the enzyme was added into the reaction mixture after the addition of 50% (w/v) trichloroacetic acid (TCA).

Protein concentration was determined by the method of Bradford using bovine serum albumin (BSA) as a protein standard.^13^

### 2.4 Three-phase partitioning (TPP) system

#### 2.4.1 Effect of salts on protease partitioning

TPP was performed using SE or ASE with protein content of 6 mg mL^−1^ according to the method of Roy and Gupta^14^ with some modifications. Firstly, TPP containing different salts, ammonium sulphate ((NH_4_)_2_SO_4_), dipotassium hydrogen phosphate (K_2_HPO_4_) and trisodium citrate (Na_3_C_6_H_5_O_7_), at different concentrations (30, 40 and 50%, w/w) was investigated by using the ratio of SE or ASE to *t*-butanol of 1.0 : 0.5 (v/v). The mixtures were mixed continuously for 3 min using a vortex mixer (G506E, Scientific Industries, USA) and then allowed to stand for 60 min before subjecting to centrifuge at 7500 × *g* for 20 min at 4 °C to facilitate the separation of phases. Each phase was carefully separated using a Pasteur pipette. The lower aqueous layer and the interfacial phase were collected and dialyzed against 50 volumes of distilled water using a dialysis bag (MW cut-off: 14 000 Da; EIDIA Co., Ltd, Tokyo, Japan) overnight at 4 °C with 4 changes of distilled water. After dialysis, the samples were measured for volume, protease activity and total protein content. TPP partitioning parameters including yield, specific activity (SA) and purification factor (PF) were calculated as follows:
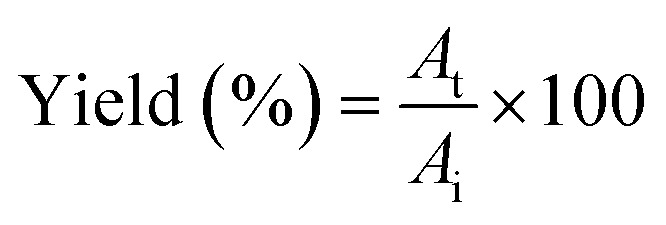
where *A*_t_ is total protease activity in the protease rich phase and *A*_i_ is the initial protease activity of SE or ASE or fraction before being partitioned.
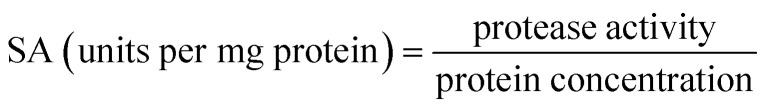

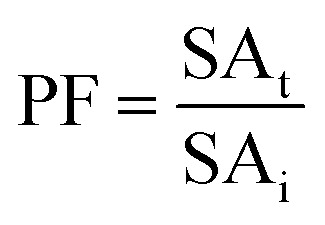
where SA_t_ is the SA of the protease rich phase and SA_i_ is the initial SA of SE or ASE or fraction before being partitioned. The system providing the highest protease recovery and purity was chosen for further study.

#### 2.4.2 Effect of organic solvents on protease partitioning

The effect of organic solvents on partitioning of protease was studied. Firstly, SE or ASE was added with different organic solvents (1-butanol and *t*-butanol) at different ratios (1.0 : 0.5, 1.0 : 1.0 and 1.0 : 1.5, w/w). Thereafter, ammonium sulphate at 40% saturation was added into the mixture. Partitioning was performed as described previously. The SE or ASE/organic solvent ratio yielding the highest enzyme recovery and purity was selected. The selected TPP fractions were used for ATPS.

### 2.5 Aqueous two-phase system (ATPS)

ATPS was prepared in 10 mL centrifuge tubes by adding the different amounts of PEG and salts together with the selected TPP fraction of SE or ASE according to the method of Kuepethkaew *et al.*^6^ and Klomklao *et al.*^8^

#### 2.5.1 Effect of salts on protease partitioning in TPP fraction

To study the effect of salts on protease partitioning in TPP fraction of SE or ASE, ATPS containing different salts, ammonium sulphate ((NH_4_)_2_SO_4_), magnesium sulfate (MgSO_4_) and trisodium citrate (Na_3_C_6_H_5_O_7_), at different concentrations (15, 20 and 25%, w/w) with 20% (w/w) PEG1000 were used. One mL of TPP fraction of SE or ASE (2 mg protein per mL) was added into the system. Distilled water was used to adjust the system to obtain the final weight of 5 g. The mixtures were mixed continuously for 3 min using a vortex mixer (G506E, Scientific Industries, USA). Phase separation was achieved by centrifugation for 10 min at 4 °C at 5000 × *g*. The top and bottom phases were carefully separated using a Pasteur pipette and the interface of each tube was discarded. Volumes of the separated phases, top and bottom phases, were measured. Aliquots from each phase were taken for protease assay and protein determination. Yield, SA and PF were then calculated. Additionally, ATPS partitioning parameters including volume ratio (*V*_R_), the partition coefficient of protein (*K*_P_) and the partition coefficient of enzyme (*K*_E_) were calculated as follows:
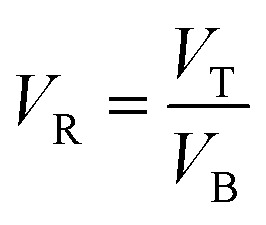
where *V*_T_ and *V*_B_ are the volume of top phase and bottom phase, respectively.
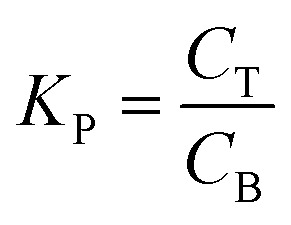
where *C*_T_ and *C*_B_ are concentrations of protein in top phase and bottom phase, respectively.
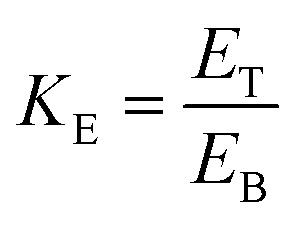
where *E*_T_ and *E*_B_ are concentrations of enzyme in top phase and bottom phase, respectively.

Based on purity and yield, the appropriate salt in ATPS rendering the most effective partitioning was selected for further study.

#### 2.5.2 Effect of MW and concentration of PEG on protease partitioning in TPP fraction

Effect of PEG1000, PEG2000 and PEG4000 at different concentrations (15, 20 and 25%, w/w) on partitioning of proteases in TPP fraction of SE or ASE was investigated in the presence of salt with the type and concentration showing the highest yield and purity. Partitioning was performed as described previously. ATPS giving the most effective partitioning of protease was chosen for further study.

### 2.6 Back extraction (BE)

BE was used to partition the protease in PEG rich phase to aqueous salt rich phase following the method of Malpiedi *et al.*^15^ and Senphan and Benjakul^9^ with a slight modification. System containing 25% PEG4000 or 25% PEG8000 in the present of Na_3_C_6_H_5_O_7_ at different final concentrations (5, 10 and 15%, w/w) were used. One gram of the selected ATPS fraction of SE or ASE was added into the prepared systems. Distilled water was used to adjust the system to obtain the final weight of 5 g. The mixtures were mixed continuously for 3 min using a vortex mixer. Phase separation was achieved by centrifugation for 10 min at 5000 × *g*. The lower aqueous layer was collected and dialyzed against 50 volumes of distilled water using a dialysis bag (MW cut-off: 14 000 Da; EIDIA Co., Ltd, Tokyo, Japan) overnight at 4 °C with 4 changes of distilled water. After dialysis, the samples were measured for enzyme activity and total protein content. Yield, SA, PF and ATPS partition parameters were calculated. BE fraction of SE or ASE with the highest purity and yield was used for further study.

### 2.7 Sodium dodecyl sulphate polyacrylamide gel electrophoresis (SDS-PAGE)

SDS-PAGE of SE, ASE and their fractionated proteases was performed using Mini-Protein II Cell apparatus (Bio-Rad Laboratories, Richmond, CA, USA) as described by Laemmli.^16^ In this study, acrylamide gel was made from 4.0% stacking and 12.0% separating gels. The SDS-PAGE procedure has been described previously.^6,8,9^

### 2.8 Storage stability of stomach protease

Protease activity of SE and ASE and their selected BE fractions (BE-SE and BE-ASE, respectively) containing 0.1% (w/v) sodium azide was monitored daily for the first 8 days and thereafter every 2 days up to 2 weeks. The storage temperature studied included −20 °C, 0 °C, 4 °C and room temperature (26–28 °C).

### 2.9 Statistical analysis

Experiments were run in triplicate using three different lots of samples. A complete randomized design was applied in this study. Analysis of variance (ANOVA) was used to analyze the data. Values of means were compared by Duncan's multiple range tests.^17^ Statistical analysis was carried out by the Statistical Package for Social Science software (SPSS 24.0, SPSS Inc., Chicago, IL, USA).

## 3. Results and discussion

### 3.1 Use of TPP for stomach proteases partitioning

#### 3.1.1 Effect of salts with different types and concentrations

**Fig. 1:**
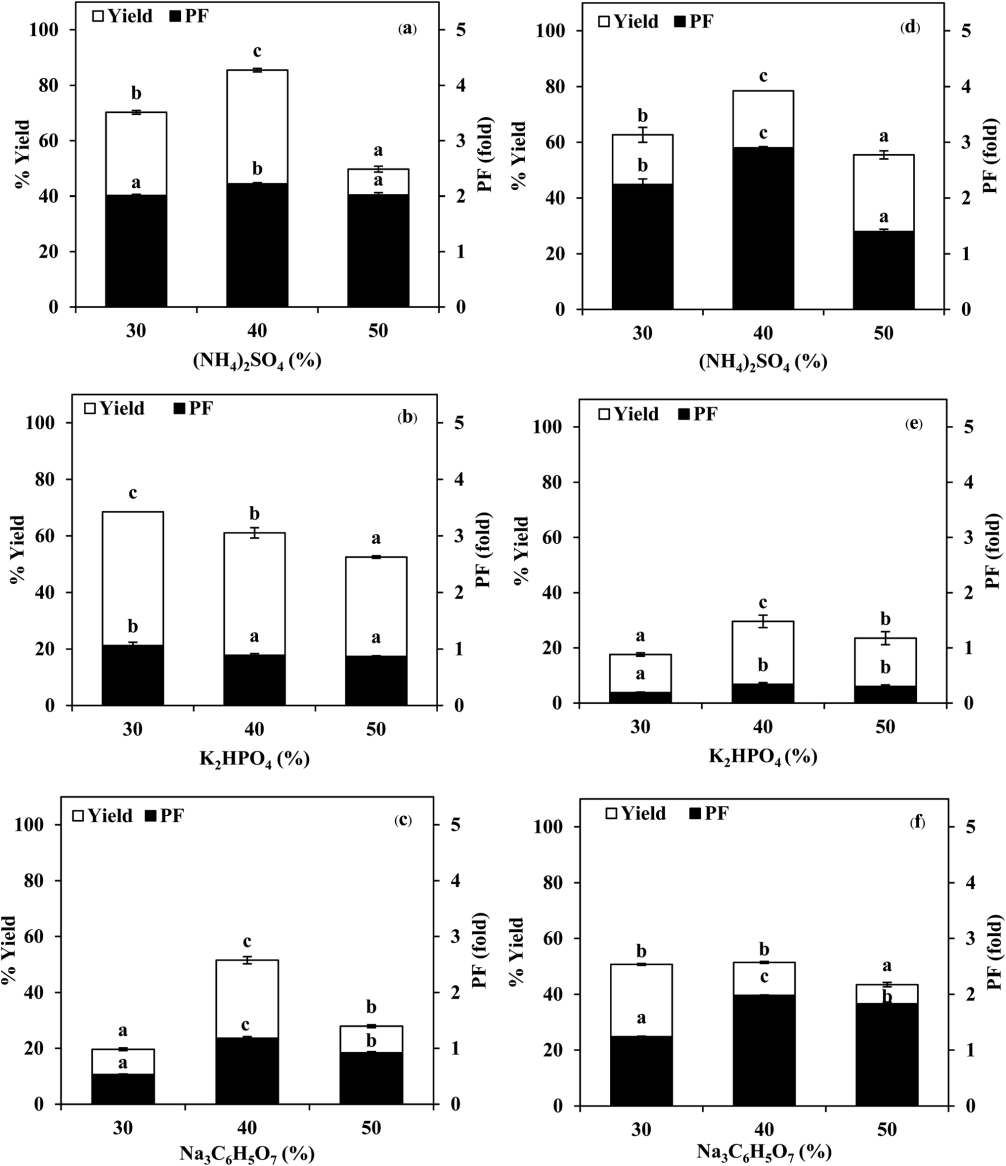
Effect of salts with different types and concentrations on the yield and purification fold of SE (a–c) and ASE (d–f). Different letters on the bar within the same parameter indicate significant differences (*p* < 0.05). Bars represent the standard deviation from triplicate determinations.

It is a pre-requisite to find the optimum kosmotropic salt concentration and type for efficient protein purification by TPP in order to precipitate the protein in the interfacial phase. The effect of salt types ((NH_4_)_2_SO_4_, Na_3_C_6_H_5_O_7_ and K_2_HPO_4_) and concentrations (30, 40 and 50%, w/w) on the partitioning parameter (yield and PF) of SE and ASE from lizardfish stomach are shown in Fig. 1. For all TPP system investigated, the proteases in both SE and ASE were partitioning predominantly in the middle phase or interphase as evidenced by negligible or no protease activity in the top and bottom phases (data not shown). The TPP system containing 40% (NH_4_)_2_SO_4_ exhibited superior partitioning affinity for both SE and ASE to those containing other salts. For SE, 40% (NH_4_)_2_SO_4_ gave the best results with 2.22-fold purification and 85.49% recovery of protease activity. The maximal yield (78.50%) and PF (2.90-fold) from the TPP system containing 40% (NH_4_)_2_SO_4_ was obtained for partitioning of ASE. The other salts (Na_3_C_6_H_5_O_7_ and K_2_HPO_4_) exhibited the lower PF and yield values of both SE and ASE. Above 40% of ammonium sulphate concentration, there is a decrease in the purification fold and yield of SE or ASE because of the protein precipitation and may be possibly due to its irreversible denaturation.^6,9^ Ammonium sulphate, is the most popular salt applied for protein salting-out as it is cheap, is readily available, is gentle on proteins, stabilizes some proteins, and due to its high solubility. From other previous studies, no evidence was found that other salts were superior or even equal to ammonium sulphate.^6,9^ Additionally, NH_4_^+^ and SO_4_^2−^ are at the ends of their respective Hofmeister series and have been shown to stabilize intermolecular interactions in macromolecules such as protein structure. Ammonium sulphate saturation is of critical importance and plays a major role in TPP as it is responsible for protein–protein interaction and precipitation. It causes protein precipitation by salting-out mechanism. The efficiency of the salting-out will first depend on the amount of ammonium sulphate and second on the ionic strength of the solution.^18^ Earlier investigations also supports that ammonium sulphate is the best salt for carrying out TPP efficiently.^6,7,9^ From the results, as the highest purity and protease recovery for both SE and ASE was achieved at 40% (NH_4_)_2_SO_4_ concentration, it was selected as optimum value for the next set of experiments.

#### 3.1.2 Effect of organic solvents with different types and ratios

**Fig. 2:**
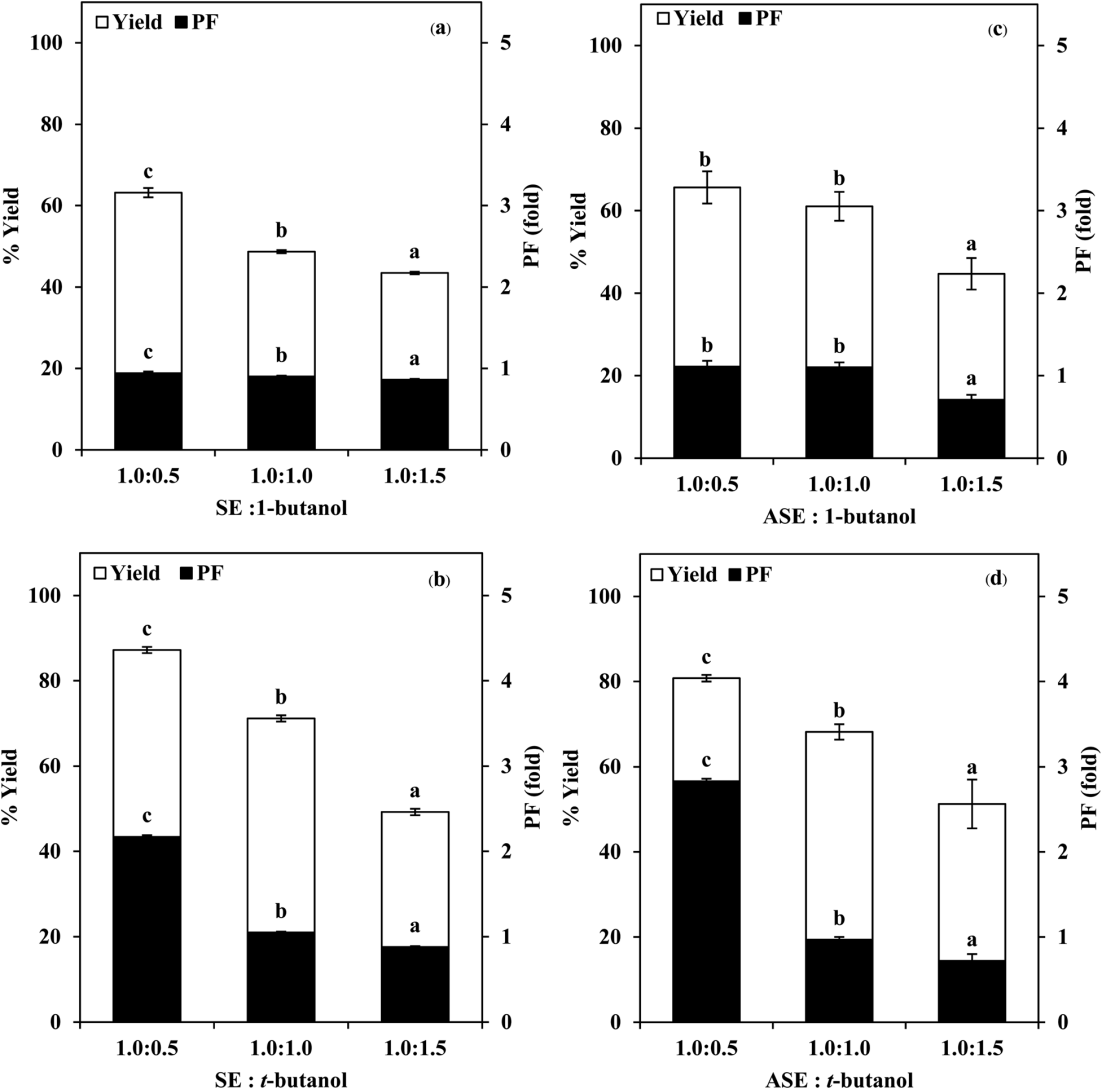
Effect of organic solvents with different types and ratios on the yield and purification fold of SE (a and b) and ASE (c and d). Different letters on the bar within the same parameter indicate significant differences (*p* < 0.05). Bars represent the standard deviation from triplicate determinations.

In this TPP based method for SE or ASE separation, *t*-butanol has been found to be consistently better than 1-butanol (Fig. 2). Due to its size and branched structure, *t*-butanol does not easily permeate inside the folded protein molecules and thus does not cause denaturation.^6^ The effect of SE or ASE volume to organic solvents on the separation of protease was also studied by varying amounts of 1-butanol and *t*-butanol to maintain the ratios as 1.0 : 0.5, 1.0 : 1.0 and 1.0 : 1.5 (w/w). From Fig. 2, it was observed that the yield and purity of both SE and ASE were highest at the 1.0 : 0.5 ratio and an increase in crude volume to organic solvents ratio has shown a significant decrease in the activity recovery and purification fold values. These results may be attributed to the synergistic effects of the increase in the concentration of organic solvents and the decrease in the saturation of ammonium sulphate.^7^ High organic solvent content may cause denaturation of the protein and hinders protein precipitation. Therefore, a ratio of 1.0 : 0.5 of crude volume to *t*-butanol was considered as optimum with resulting protease recovery for SE and ASE of 87.22 and 80.77%, respectively. The crude extract to *t*-butanol ratio of 1.0 : 0.5 in the present of 50% (NH_4_)_2_SO_4_ was the optimal condition for the proteases partitioning from the viscera of farmed giant catfish.^7^ Senphan and Benjakul^9^ found that the optimum TPP condition of protease from hepatopancreas of Pacific white shrimp was 30% (NH_4_)_2_SO_4_ along with 1.0 : 1.0 ratio of *t*-butanol to crude protease extract provided the enzyme recovery 76.0%.

### 3.2 Use of ATPS for stomach proteases partitioning

#### 3.2.1 Effect of salts with different types and concentrations

The partitioning of protease from TPP fraction of both SE and ASE was carried out using several biphasic systems of 20% PEG1000 containing different salts including (NH_4_)_2_SO_4_, MgSO_4_ and Na_3_C_6_H_5_O_7_ at different concentrations (15, 20 and 25%, w/w) (Tables 1 and 2, respectively). After phase separation, two phases were obtained, PEG-rich top phase and salt-rich lower phase. However, no phase separation was found in the system containing 15% MgSO_4_ for SE partitioning. For ASE partitioning, the system containing 15% MgSO_4_ and 15% Na_3_C_6_H_5_O_7_ could not separate the phases. It is probably due to the amount of both salts were not sufficient to generate the two-phase formation in this partitioning of proteases from lizardfish stomach. For all ATPS tested, the proteases in both SE and ASE were partitioned predominantly in the PEG-rich top phase, principally those with hydrophobic properties.^6,8,9^ The recovery of proteases in SE and ASE from the opposite phase (lower phase) was relatively low. Generally, negatively charged proteins prefer the upper phase in PEG–salt systems, while positively charged proteins normally partition selectively to the bottom phase.^8,9^ Therefore, stomach proteases from lizardfish, both non-activated and activated, partitioned in the top phase might be negative charged. These results might be explained by the acceptance of enzyme polyanions by PEG-rich phase in ATPS that occurred above pI. For SE, a phase system containing 20% PEG1000 and 15% Na_3_C_6_H_5_O_7_ gave the highest SA (510.11 units per mg protein), PF (4.34-fold) and yield (83.96%) (*p* < 0.05). For ASE, ATPS containing 15% (NH_4_)_2_SO_4_ exhibited the highest yield but it was not a significant difference from the system containing 20% PEG1000 and 20% Na_3_C_6_H_5_O_7_ (*p* > 0.05). Also, the maximum SA and PF from the system containing 20% PEG1000 and 20% Na_3_C_6_H_5_O_7_ were obtained for partitioning of ASE. The type and concentration of salts, and the ratio between each ion concentration in both phases is very important when partitioning charged molecules in ATPS. This unequivocally indicates that sodium citrate is more effective in accommodating undesirable proteins in the bottom phase than the other salts. According to the Hofmeister series, citrate is both anti-chaotropic anions that strongly interact and structure water, promoting the salting out and stabilization of macromolecules in solution.^19^ Pérez *et al.*^20^ reported that the main advantages of PEG/sodium citrate systems are the biodegradability and non-toxicity of the citrate anion when comparing with the high eutrophication potential of other salt ions. Thus, the systems containing 15% or 20% Na_3_C_6_H_5_O_7_ were appropriate of protease in SE and ASE, respectively.

**Table 1:** Effect of phase composition in PEG1000–salt ATPS on partitioning of protease (SE) from lizardfish stomach^a^

Phase composition (%, w/w)	*V*_R_	*K*_P_	*K*_E_	SA	PF		Yield (%)	
					SE^b^	TPP^c^	SE^b^	TPP^c^
20% PEG1000–15% (NH_4_)_2_SO_4_	0.71 ± 0.03^d^	7.26 ± 0.10^e^	30.95 ± 2.48^e^	496.03 ± 39.87^b^	4.22 ± 0.34^b^	1.86 ± 0.15^b^	62.74 ± 5.04^c^	71.87 ± 5.77^c^
20% PEG1000–20% (NH_4_)_2_SO_4_	0.50 ± 0.02^b^	5.92 ± 0.05^c^	6.79 ± 0.22^b^	460.07 ± 15.02^b^	3.91 ± 0.12^b^	1.72 ± 0.06^b^	50.48 ± 1.65^b^	57.83 ± 1.90^b^
20% PEG1000–25% (NH_4_)_2_SO_4_	0.40 ± 0.01^a^	4.60 ± 0.04^a^	0.95 ± 0.04^a^	356.83 ± 14.34^a^	3.04 ± 0.12^a^	1.34 ± 0.06^a^	32.41 ± 1.30^a^	37.12 ± 1.49^a^
20% PEG1000–15% MgSO_4_	ns	ns	ns	ns	ns	ns	ns	ns
20% PEG1000–20% MgSO_4_	0.89 ± 0.01^e^	9.69 ± 0.04^g^	26.65 ± 3.18^d^	400.32 ± 47.74^a^	3.41 ± 0.40^a^	1.50 ± 0.18^a^	57.61 ± 6.87^c^	66.01 ± 7.87^c^
20% PEG1000–25% MgSO_4_	0.65 ± 0.02^c^	6.32 ± 0.02^d^	20.25 ± 1.12^c^	353.31 ± 19.52^a^	3.01 ± 0.17^a^	1.32 ± 0.07^a^	35.57 ± 1.97^a^	40.75 ± 2.25^a^
20% PEG1000–15% Na_3_C_6_H_5_O_7_	1.02 ± 0.02^f^	5.66 ± 0.02^b^	56.49 ± 4.32^g^	510.11 ± 39.00^b^	4.34 ± 0.46^b^	1.91 ± 0.14^b^	83.96 ± 6.42^d^	96.18 ± 7.35^d^
20% PEG1000–20% Na_3_C_6_H_5_O_7_	0.64 ± 0.02^c^	7.21 ± 0.14^e^	45.80 ± 1.54^f^	458.04 ± 15.36^b^	3.89 ± 0.13^b^	1.72 ± 0.06^b^	80.47 ± 2.70^d^	92.17 ± 3.09^d^
20% PEG1000–25% Na_3_C_6_H_5_O_7_	0.47 ± 0.02^b^	7.98 ± 0.05^f^	63.39 ± 1.31^h^	372.42 ± 7.70^a^	3.17 ± 0.06^a^	1.39 ± 0.03^a^	77.10 ± 1.59^d^	88.32 ± 1.82^d^

a Means ± SD from the triplicate determinations.

b Values were expressed, relative to those of SE.

c Values were expressed, relative to those of TPP fraction.

**Table 2:** Effect of phase composition in PEG1000–salt ATPS on partitioning of acidified protease (ASE) from lizardfish stomach^a^

Phase composition (%, w/w)	*V*_R_	*K*_P_	*K*_E_	SA	PF		Yield (%)	
					ASE^b^	TPP^c^	ASE^b^	TPP^c^
20% PEG1000–15% (NH_4_)_2_SO_4_	0.74 ± 0.02^d^	8.84 ± 0.03^f^	51.18 ± 1.19^e^	364.91 ± 8.52^b^	4.34 ± 0.10^b^	1.53 ± 0.04^b^	77.54 ± 1.81^c^	96.11 ± 2.24^c^
20% PEG1000–20% (NH_4_)_2_SO_4_	0.49 ± 0.03^b^	6.44 ± 0.01^d^	10.72 ± 0.58^a^	356.32 ± 19.26^b^	4.24 ± 0.23^b^	1.50 ± 0.08^b^	62.96 ± 3.40^b^	78.04 ± 4.22^b^
20% PEG1000–25% (NH_4_)_2_SO_4_	0.40 ± 0.01^a^	5.13 ± 0.04^b^	20.21 ± 1.78^b^	347.11 ± 30.56^b^	4.13 ± 0.37^b^	1.46 ± 0.13^b^	53.58 ± 4.72^a^	66.41 ± 5.85^a^
20% PEG1000–15% MgSO_4_	ns	ns	ns	ns	ns	ns	ns	ns
20% PEG1000–20% MgSO_4_	0.87 ± 0.01^e^	13.20 ± 0.24^g^	11.47 ± 1.09^a^	277.27 ± 26.34^a^	3.30 ± 0.31^a^	1.17 ± 0.11^a^	65.16 ± 6.19^b^	80.76 ± 7.67^b^
20% PEG1000–25% MgSO_4_	0.64 ± 0.03^c^	7.60 ± 0.05^e^	24.53 ± 2.57^c^	340.34 ± 35.63^b^	4.05 ± 0.42^b^	1.43 ± 0.15^b^	60.40 ± 6.32^ab^	74.86 ± 7.84^ab^
20% PEG1000–15% Na_3_C_6_H_5_O_7_	ns	ns	ns	ns	ns	ns	ns	ns
20% PEG1000–20% Na_3_C_6_H_5_O_7_	0.62 ± 0.02^c^	5.99 ± 0.10^c^	66.32 ± 2.41^f^	383.73 ± 13.91^b^	4.56 ± 0.16^b^	1.62 ± 0.06^b^	75.37 ± 2.73^c^	93.41 ± 3.39^c^
20% PEG1000–25% Na_3_C_6_H_5_O_7_	0.49 ± 0.01^b^	4.86 ± 0.13^a^	45.04 ± 2.86^d^	348.80 ± 22.17^b^	4.15 ± 0.26^b^	1.47 ± 0.09^b^	59.72 ± 3.80^ab^	74.02 ± 4.70^ab^

a Means ± SD from the triplicate determinations.

b Values were expressed, relative to those of ASE.

c Values were expressed, relative to those of TPP fraction.

From the result, the *V*_R_ of all ATPS investigated of both SE and ASE was decreased when salt concentration increased. Enhancement of salt quantity provided a higher proportion of salt-rich bottom phase leading to practically reduced *V*_R_. The distribution of the protein and proteases in ATPS are reported by *K*_P_ and *K*_E_, respectively. The distribution of protein in ATPS of SE and ASE likely depended on the differences in surface charge of protease and others protein contaminants, which were governed by different salts.^3,8^ High in *K*_P_ values indicating most of proteins were more partitioning to the top phase, while the high in *K*_E_ implying only the target enzyme was partitioned to the top phase. Generally, ATPS with 20% and 25% Na_3_C_6_H_5_O_7_ showed higher *K*_E_. In general, increasing salts concentration for both SE and ASE partitioning resulted in lower *K*_P_. Salts might increase the migration of the proteins to the lower phase by electrostatic repulsion effects. Tables 1 and 2 also exhibited that a less protease activity recovery was observed with increasing salt concentration. It can be found that increase in salt quantity provided the salting-out effect.^8^ Ketnawa *et al.*^21^ reported that the highest purification factor and yield in partitioning of alkaline protease from farmed giant catfish (*Pangasianodon gigas*) viscera was obtained by using 15% PEG2000–15% sodium citrate.

#### 3.2.2 Effect of PEG with different MW and concentrations

The molecular weight of PEG affects its distribution in the two phases and polymer–protein interaction.^22^ In order to make a selection of suitable molecular weight of PEG for purification and recovery of proteases from TPP fraction of both SE and ASE, ATPS was performed with different molecular weights (PEG1000, 2000 and 4000) at different concentrations (15, 20 and 25%, w/w) in the presence of 15% or 20% Na_3_C_6_H_5_O_7_ for SE and ASE, respectively (Tables 3 and 4). The protease partitionings were strongly dependent on the MW and concentration of PEG. The enzyme partitioned preferentially towards the PEG-rich phase. From the result, ATPS of 20% PEG1000–15% Na_3_C_6_H_5_O_7_ gave the highest PF (4.48-fold) with 82.97% yield for partitioning of protease in SE, while the 15% PEG1000–20% Na_3_C_6_H_5_O_7_ afforded the greatest PF (5.13-fold) with 77.52% yield for ATPS in partitioning protease in ASE. When the same PEG concentration was applied, the phase volume ratio of SE and ASE generally decreased when MW of PEG increased. However, at the same PEG molecular weight, *V*_R_ of both SE and ASE increased with increasing PEG concentration. Similar trends were generally observed for *K*_P_ except for partitioning of protease in ASE using the ATPS containing 25% PEG1000–20% Na_3_C_6_H_5_O_7_ exhibiting the lower *K*_P_. For *K*_E_ of both SE and ASE, the higher of *K*_E_ values observed for PEG1000 suggests an increase in PEG–protein interactions. The *K*_P_ and *K*_E_ decreased with increasing PEG molecular weight, because of the reduction in space for SE and ASE in the top phase, as a result the proteins and enzymes tend to partition to the bottom phase, which is inferred as “volume exclusion effect”.^23^ In general, increasing the MW of the phase polymers resulting in the distribution of biomolecule towards more strongly into the other phase as the repulsive interactions between the polymer and biomolecules become stronger. When the same molecules are added into phase system with different MW of polymer, their partition coefficient decrease as MW increase.^5^ Additionally, use of the higher MW of PEG gave a lower yield of SE and ASE recovered, compared with the lower MW. For ATPS formed by PEG of low MW (600–3350 kDa), the proteins transfer to the top phase is enthalpically driven, mainly due to a strong interaction between PEG and the protein.^3,6,9^ Klomklao *et al.*^8^ found that for the production of proteinase from yellowfin tuna by ATPS, the lower MW of PEG1000 produced a higher yield than PEG4000. Senphan and Benjakul^9^ also reported that a system consisting of PEG1000 for proteases from Pacific white shrimp hepatopancreas production achieved a high yield compared with that of PEG2000 and 4000. Therefore, PEG1000 was a suitable polymer for partitioning of protease in TPP fraction of both SE and ASE as indicated by the higher PF and yield than PEG with higher MWs.

**Table 3:** Effect of PEG-molecular mass and concentration in a PEG–Na_3_C_6_H_5_O_7_ ATPS on partitioning of protease (SE) from lizardfish stomach^a^

Phase composition (%, w/w)	*V*_R_	*K*_P_	*K*_E_	SA	PF		Yield (%)	
					SE^b^	TPP^c^	SE^b^	TPP^c^
15% PEG1000–15% Na_3_C_6_H_5_O_7_	ns	ns	ns	ns	ns	ns	ns	ns
20% PEG1000–15% Na_3_C_6_H_5_O_7_	1.01 ± 0.0^1f^	6.74 ± 0.21^f^	51.16 ± 0.91^f^	526.94 ± 9.38^c^	4.48 ± 0.08^c^	1.97 ± 0.04^c^	82.97 ± 1.48^e^	95.04 ± 1.70^e^
25% PEG1000–15% Na_3_C_6_H_5_O_7_	1.07 ± 0.01^g^	6.91 ± 0.23^f^	30.23 ± 2.89^e^	499.79 ± 47.79^ab^	4.25 ± 0.41^bc^	1.87 ± 0.18^bc^	65.38 ± 6.25^d^	74.89 ± 7.06^d^
15% PEG2000–15% Na_3_C_6_H_5_O_7_	0.67 ± 0.01^c^	3.14 ± 0.01^b^	6.49 ± 0.51^bc^	300.09 ± 23.64^a^	2.55 ± 0.20^a^	1.12 ± 0.09^a^	27.21 ± 2.15^b^	31.17 ± 2.45^b^
20% PEG2000–15% Na_3_C_6_H_5_O_7_	0.82 ± 0.01^d^	4.52 ± 0.04^d^	11.20 ± 0.85^d^	501.78 ± 37.90^ab^	4.27 ± 0.32^bc^	1.88 ± 0.14^bc^	51.47 ± 3.89^c^	58.96 ± 4.45^c^
25% PEG2000–15% Na_3_C_6_H_5_O_7_	0.94 ± 0.02^e^	5.18 ± 0.04^e^	7.43 ± 0.71^c^	439.73 ± 42.18^b^	3.74 ± 0.36^b^	1.65 ± 0.16^b^	49.20 ± 4.72^c^	56.36 ± 5.41^c^
15% PEG4000–15% Na_3_C_6_H_5_O_7_	0.47 ± 0.02^a^	2.58 ± 0.01^a^	2.83 ± 0.51^a^	249.74 ± 44.74^a^	2.12 ± 0.38^a^	0.94 ± 0.17^a^	17.95 ± 3.21^a^	20.57 ± 3.68^a^
20% PEG4000–15% Na_3_C_6_H_5_O_7_	0.54 ± 0.01^b^	3.27 ± 0.03^b^	4.91 ± 0.31^b^	509.94 ± 31.64^c^	4.34 ± 0.27^c^	1.91 ± 0.12^c^	44.45 ± 2.76^c^	50.92 ± 3.16^c^
25% PEG4000–15% Na_3_C_6_H_5_O_7_	0.69 ± 0.01^c^	4.27 ± 0.01^c^	7.92 ± 0.60^c^	435.41 ± 33.26^b^	3.70 ± 0.28^b^	1.63 ± 0.12^b^	46.01 ± 3.51^c^	52.71 ± 4.03^c^

a Means ± SD from the triplicate determinations.

b Values were expressed, relative to those of SE.

c Values were expressed, relative to those of TPP fraction.

**Table 4:** Effect of PEG-molecular mass and concentration in a PEG–Na_3_C_6_H_5_O_7_ ATPS on partitioning of acidified protease (ASE) from lizardfish stomach^a^

Phase composition (%, w/w)	*V*_R_	*K*_P_	*K*_E_	SA	PF		Yield (%)	
					ASE^b^	TPP^c^	ASE^b^	TPP^c^
15% PEG1000–20% Na_3_C_6_H_5_O_7_	0.50 ± 0.03^b^	5.18 ± 0.01^d^	29.24 ± 0.99^g^	431.33 ± 14.63^d^	5.13 ± 0.17^d^	1.81 ± 0.06^d^	77.52 ± 2.63^f^	96.09 ± 3.26^f^
20% PEG1000–20% Na_3_C_6_H_5_O_7_	0.70 ± 0.01^d^	6.06 ± 0.02^f^	65.81 ± 0.97^h^	390.19 ± 5.75^c^	4.64 ± 0.07^c^	1.64 ± 0.03^c^	74.79 ± 1.10^ef^	92.69 ± 1.37^ef^
25% PEG1000–20% Na_3_C_6_H_5_O_7_	0.80 ± 0.01^e^	4.40 ± 0.03^g^	22.53 ± 0.90^f^	386.97 ± 15.48^c^	4.60 ± 0.18^c^	1.63 ± 0.07^c^	59.73 ± 2.39^c^	74.03 ± 2.96^c^
15% PEG2000–20% Na_3_C_6_H_5_O_7_	0.42 ± 0.01^a^	4.03 ± 0.02^b^	9.52 ± 0.16^cd^	410.61 ± 6.77^d^	4.88 ± 0.08^d^	1.73 ± 0.03^d^	59.49 ± 1.01^c^	73.74 ± 1.21^c^
20% PEG2000–20% Na_3_C_6_H_5_O_7_	0.63 ± 0.02^c^	5.22 ± 0.07^d^	16.04 ± 0.24^e^	416.63 ± 6.10^d^	4.96 ± 0.08^d^	1.75 ± 0.03^d^	72.91 ± 1.07^e^	90.37 ± 1.32^e^
25% PEG2000–20% Na_3_C_6_H_5_O_7_	0.68 ± 0.01^cd^	5.44 ± 0.02^e^	9.96 ± 0.25^d^	312.17 ± 7.86^b^	3.71 ± 0.09^b^	1.31 ± 0.03^b^	56.58 ± 1.43^bc^	70.13 ± 1.76^bc^
15% PEG4000–20% Na_3_C_6_H_5_O_7_	0.36 ± 0.04^a^	3.12 ± 0.02^a^	4.77 ± 0.14^a^	428.20 ± 12.23^d^	5.09 ± 0.15^d^	1.80 ± 0.05^d^	53.37 ± 1.52^b^	66.14 ± 1.89^b^
20% PEG4000–20% Na_3_C_6_H_5_O_7_	0.49 ± 0.04^b^	4.15 ± 0.12^c^	8.83 ± 0.21^c^	425.52 ± 9.98^d^	5.06 ± 0.12^d^	1.79 ± 0.04^d^	65.20 ± 1.53^d^	80.81 ± 1.90^d^
25% PEG4000–20% Na_3_C_6_H_5_O_7_	0.62 ± 0.05^c^	5.14 ± 0.10^d^	6.81 ± 0.57^b^	218.21 ± 18.28^a^	2.60 ± 0.22^a^	0.92 ± 0.08^a^	38.68 ± 3.24^a^	47.94 ± 4.01^a^

a Means ± SD from the triplicate determinations.

b Values were expressed, relative to those of ASE.

c Values were expressed, relative to those of TPP fraction.

### 3.3 Use of BE for stomach proteases partitioning

To separate the protease in the PEG-rich phase after ATPS separation, different methods can be applied.^3^ The target protease can be separated from PEG by back-extraction, in which a fresh salt phase is added to the collected PEG-rich phase containing the target enzyme. By changing the system conditions, target protease can be directed to the salt phase. Thereafter, salt can be removed by dialysis or membrane filtration.^9,15^ Therefore, protease separation from PEG of SE and ASE was investigated. In the present investigation, ATPS containing 25% PEG4000 and 8000 in the presence of Na_3_C_6_H_5_O_7_ at varying concentrations were used for back extraction as displayed in Tables 5 and 6. For both SE and ASE, BE using ATPS containing 25% PEG8000 and 5% Na_3_C_6_H_5_O_7_ produced the highest SA and PF (*p* < 0.05). Under the optimal conditions of the BE system, the highest purity by 7.85-fold with a recovered activity of 54.35% and 8.41-fold with a recovered activity of 64.81% was obtained for SE and ASE, respectively, compared with SE and ASE. In the presence of 5, 10 or 15% Na_3_C_6_H_5_O_7_, PEG8000 exhibited higher SA and PF than PEG4000. PEG of the highest MW (PEG8000) excludes the protein from the top phase, driven by an entropically unfavourable term.^6,9^ BE for proteases from Pacific white shrimp hepatopancreas was achieved using high MW PEG (25% PEG8000).^9^ From the results, ATPS containing 25% PEG8000 and 5% Na_3_C_6_H_5_O_7_ was shown to be the effective BE to transfer proteases of SE and ASE to the salt phase. Those phase systems exhibiting the highest PF with appropriate yield for SE or ASE were selected for further investigation and those fractions were referred to “BE-SE” and “BE-ASE”, respectively.

**Table 5:** BE for partitioning of protease (SE) from lizardfish stomach as affected by PEG with different molecular weight and Na_3_C_6_H_5_O_7_ at various concentrations^a^

Phase composition (%, w/w)	*V*_R_	*K*_P_	*K*_E_	SA	PF		Yield (%)	
					SE^b^	TPP^c^	SE^b^	TPP^c^
25% PEG4000–5% Na_3_C_6_H_5_O_7_	5.93 ± 0.11^d^	0.32 ± 0.01^e^	1.56 ± 0.01^e^	583.01 ± 6.74^b^	4.96 ± 0.06^b^	2.18 ± 0.03^b^	24.82 ± 0.29^a^	28.43 ± 0.33^a^
25% PEG4000–10% Na_3_C_6_H_5_O_7_	1.86 ± 0.08^b^	0.14 ± 0.01^b^	0.64 ± 0.03^c^	559.90 ± 2.68^ab^	4.76 ± 0.02^ab^	2.10 ± 0.01^ab^	56.37 ± 0.27^c^	64.57 ± 0.31^c^
25% PEG4000–15% Na_3_C_6_H_5_O_7_	1.21 ± 0.03^a^	0.09 ± 0.01^a^	0.36 ± 0.07^a^	546.50 ± 31.65^a^	4.65 ± 0.27^a^	2.05 ± 0.12^a^	72.01 ± 4.17^d^	82.48 ± 4.78^d^
25% PEG8000–5% Na_3_C_6_H_5_O_7_	6.24 ± 0.07^e^	0.28 ± 0.01^d^	0.73 ± 0.02^d^	922.43 ± 16.73^d^	7.85 ± 0.14^d^	3.46 ± 0.06^d^	54.35 ± 0.98^bc^	62.26 ± 1.13^bc^
25% PEG8000–10% Na_3_C_6_H_5_O_7_	2.34 ± 0.01^c^	0.15 ± 0.01^c^	0.58 ± 0.02^c^	624.27 ± 27.26^c^	5.31 ± 0.24^c^	2.34 ± 0.10^c^	52.73 ± 2.30^bc^	60.40 ± 2.64^bc^
25% PEG8000–15% Na_3_C_6_H_5_O_7_	2.36 ± 0.02^c^	0.14 ± 0.01^b^	0.46 ± 0.08^b^	591.69 ± 10.78^bc^	5.03 ± 0.09^bc^	2.22 ± 0.04^bc^	51.49 ± 0.94^b^	58.99 ± 1.08^b^

a Means ± SD from the triplicate determinations.

b Values were expressed, relative to those of SE.

c Values were expressed, relative to those of TPP fraction.

**Table 6:** BE for partitioning of acidified protease (ASE) from lizardfish stomach as affected by PEG with different molecular weight and Na_3_C_6_H_5_O_7_ at various concentrations^a^

Phase composition (%, w/w)	*V*_R_	*K*_P_	*K*_E_	SA	PF		Yield (%)	
					ASE^b^	TPP^c^	ASE^b^	TPP^c^
25% PEG4000–5% Na_3_C_6_H_5_O_7_	6.92 ± 0.16^e^	0.79 ± 0.02^f^	3.58 ± 0.05^e^	485.52 ± 30.71^bc^	5.77 ± 0.36^bc^	2.04 ± 0.13^bc^	20.69 ± 1.31^a^	25.64 ± 1.62^a^
25% PEG4000–10% Na_3_C_6_H_5_O_7_	2.59 ± 0.21^c^	0.30 ± 0.01^c^	1.67 ± 0.05^c^	390.83 ± 33.17^a^	4.65 ± 0.40^a^	1.64 ± 0.14^a^	36.71 ± 3.11^c^	45.50 ± 3.86^bc^
25% PEG4000–15% Na_3_C_6_H_5_O_7_	1.43 ± 0.03^a^	0.16 ± 0.01^a^	0.83 ± 0.03^a^	445.94 ± 16.27^ab^	5.30 ± 0.20^ab^	1.88 ± 0.07^ab^	61.48 ± 2.24^d^	67.21 ± 12.94^d^
25% PEG8000–5% Na_3_C_6_H_5_O_7_	1.74 ± 0.06^ab^	0.27 ± 0.01^b^	0.83 ± 0.02^a^	707.48 ± 30.33^d^	8.41 ± 0.36^d^	2.98 ± 0.13^d^	64.18 ± 2.75^d^	79.55 ± 3.41^e^
25% PEG8000–10% Na_3_C_6_H_5_O_7_	2.02 ± 0.03^b^	0.33 ± 0.01^d^	1.32 ± 0.02^b^	529.57 ± 77.74^c^	6.30 ± 0.92^c^	2.22 ± 0.33^c^	41.53 ± 6.10^c^	51.46 ± 7.55^c^
25% PEG8000–15% Na_3_C_6_H_5_O_7_	6.16 ± 0.26^d^	0.60 ± 0.01^e^	2.34 ± 0.04^d^	524.55 ± 35.31^c^	6.24 ± 0.42^c^	2.21 ± 0.15^c^	28.91 ± 1.95^b^	35.83 ± 2.41^ab^

a Means ± SD from the triplicate determinations.

b Values were expressed, relative to those of ASE.

c Values were expressed, relative to those of TPP fraction.

### 3.4 Protein pattern of stomach proteases partitioned with combined partitioning system

**Fig. 3:**
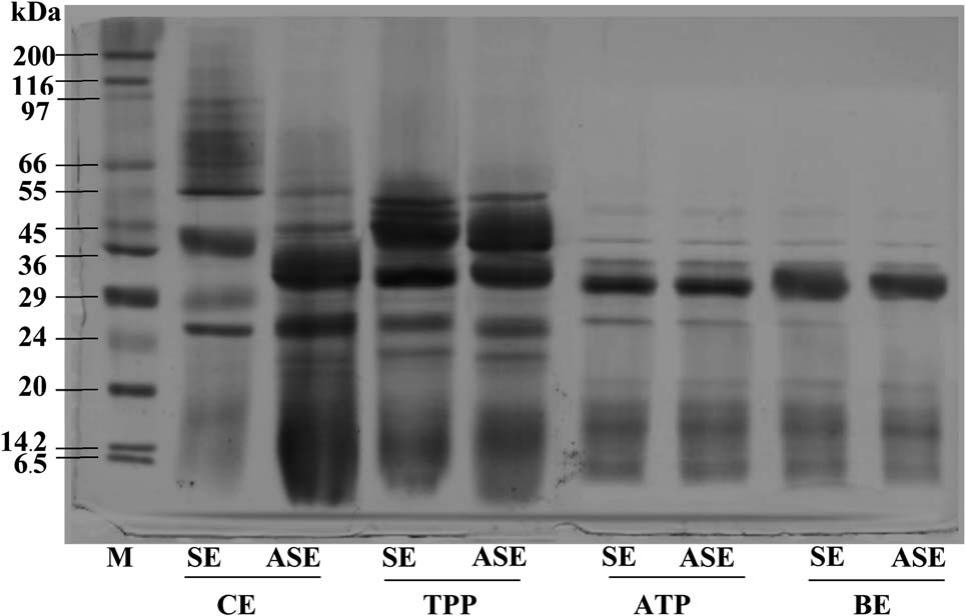
SDS-PAGE patterns of stomach extract (SE) and acidified stomach extract (ASE), and their TPP, ATPS and BE fractions. M: molecular weight standard, SE: stomach extract, ASE: acidified stomach extract, TPP: TPP fraction (the interphase of TPP containing SE or ASE to *t*-butanol ratio of 1.0 : 0.5 in the presence of 40% (NH_4_)_2_SO_4_), ATPS: ATPS fraction (the top phase of 20% PEG1000–15% Na_3_C_6_H_5_O_7_ and 15% PEG1000–20% Na_3_C_6_H_5_O for SE and ASE, respectively), BE: back extraction fraction (the bottom phase of 25% PEG8000–5% Na_3_C_6_H_5_O_7_ for SE and ASE).

The purity of protease from the lizardfish stomach after the combined partitioning system process was determined using SDS-PAGE. As can be observed in Fig. 3, both SE and ASE contained a variety of proteins with different MW. SE contained proteins with MW of 39, 29 and 26 kDa as the major proteins. After the acidification process, some protein contaminants were removed and the major band with MW of 32 and 26 kDa was found. Also, a large number of contaminating proteins were removed after partitioning with TPP, ATPS and BE, particularly proteins with higher and lower MW. As a result, a higher purity of proteases was obtained. A greater intensity of the major protein band (32 kDa) was obtained in both SE and ASE after BE process, in comparison with that observed in SE and ASE. The migration of major protein band of SE to lower molecular weight positions after acidification or ATPS partitioning might be because of the autocatalytic activation of pepsinogen to pepsin under acidic conditions and with ATPS systems, respectively. Based on our previous investigation, the aspartic protease, most likely pepsin was the major protease found in lizardfish stomachs.^10^ Generally, pepsins from marine animals were reported to have molecular weights ranging from 27 to 42 kDa.^24^ Compared with pepsin, pepsinogen contains an additional 44 amino acids, but when exposed to the hydrochloric acid (HCl) present in gastric juice (pH of 1.5–2.0), the 44 amino acids are proteolytically removed in an autocatalytic way to activate it to pepsin.^4^ From these results, the major protein band with the MW of 32 kDa was found after BE process of both SE and ASE. Therefore, the electrophoretic results clearly showed that this TPP system in combination with ATPS and BE substantially primarily purified the protease from the lizardfish stomach.

### 3.5 Storage stability of stomach proteases partitioned with combined partitioning system

**Fig. 4:**
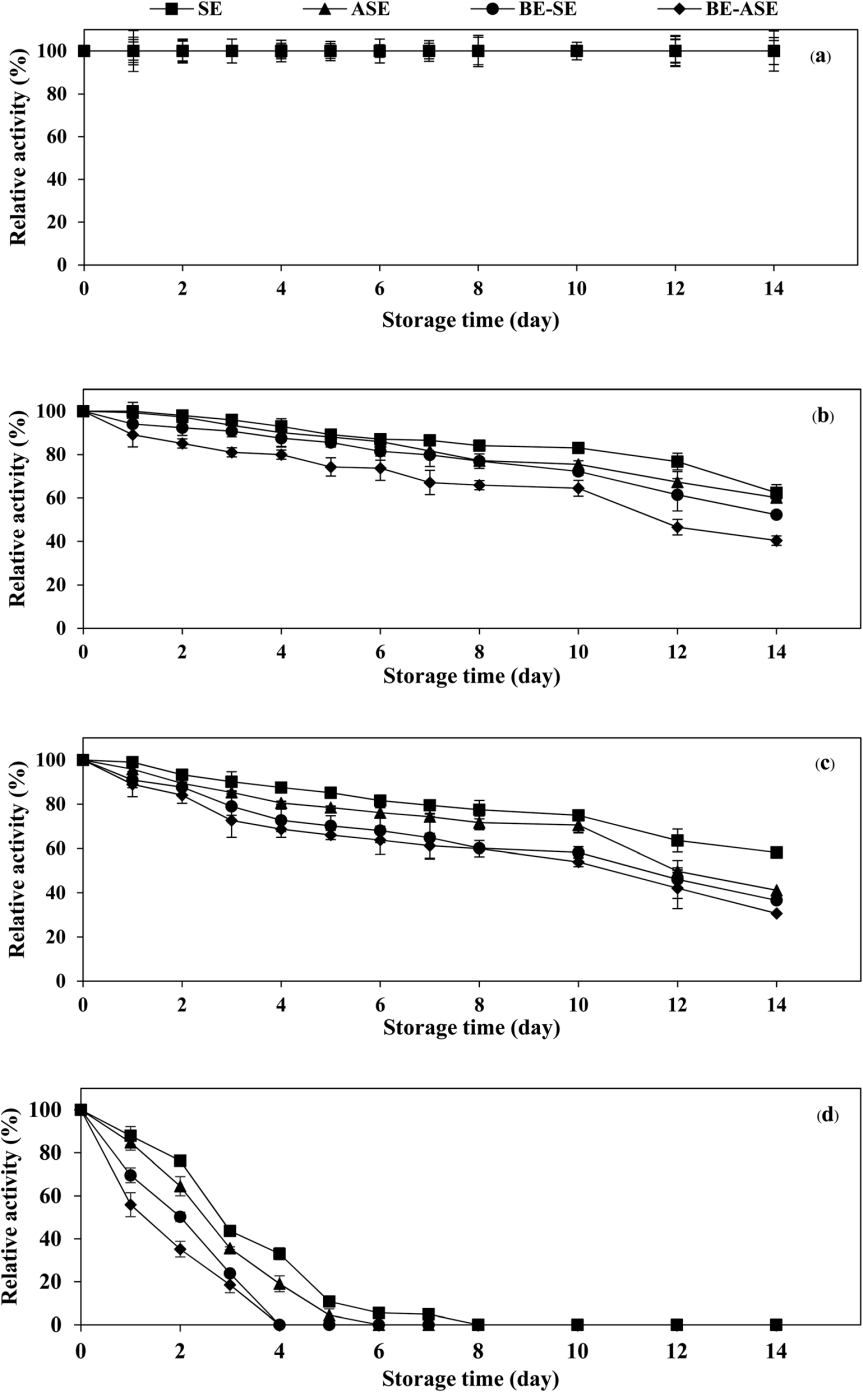
Changes in protease activity of stomach extract (SE) and acidified stomach extract (ASE), and their BE fractions (BE-SE and BE-ASE) during storage at −20 °C (a), 0 °C (b), 4 °C (c) and room temperature (d). Bars represent the standard deviation from triplicate determinations.

The stability of the enzyme is of great importance for the economy of their industrial application. Temperature is an important limiting factor for the storage of enzymes. Fig. 4 displays the changes in protease activity of SE and ASE as well as their BE fractions during storage at different temperatures (−20, 0, 4 °C and room temperature) up to 2 weeks. All samples exhibited greater stability at −20 °C. Freezing at −20 or −80 °C is the more common form of frozen protein storage. SE and ASE were quite stable for 14 days of storage at 0 and 4 °C. Nevertheless, at 0 and 4 °C, the stability of BE-SE and BE-ASE was lower as evidenced by a continuous decline in protease activity with increasing time of storage. The highest decline in protease activity was found when kept at ambient temperature. At room temperature, SE showed good stability compared to the other samples. At day 4 of storage at room temperature, for ASE, BE-SE and BE-ASE, more than 80% of the original activity was lost. The decline in protease activity noticed for all samples at room temperature may be due to increased degradation of enzyme active site with increased temperature. Storage at room temperature often leads to protein degradation and/or inactivity. The decrease in activity was higher when stored for a longer duration at higher temperatures. The least activity was observed when kept for 8 days at room temperature. A similar result was observed in the case of protease from albacore tuna (*Thunnus alalunga*) stored at room temperature.^3^ In general, enzymes are proteins and undergo essentially irreversible denaturation at temperatures above those to which they are ordinarily exposed in the environment. The results suggest that the stability of protease from lizardfish stomachs most likely depended on the form of enzyme and storage temperature. Also, low temperature, especially −20 °C was the ideal storage condition for the preservation of proteases from the lizardfish stomach.

## 4. Conclusion

The combined partitioning systems including TPP, ATPS, and BE effectively recovered and partitioned the proteases from the lizardfish stomach. TPP with SE or ASE to *t*-butanol ratio of 1.0 : 0.5 and 40% (NH_4_)_2_SO_4_ was firstly implemented. Both TPP fractions were further subjected to ATPS. For ATPS studied, the optimum condition for partitioning of SE and ASE was 20% PEG1000–15% Na_3_C_6_H_5_O_7_ and 15% PEG1000–20% Na_3_C_6_H_5_O_7_, respectively, and BE including 25% PEG8000–5% Na_3_C_6_H_5_O_7_ was applied in last step for both samples. The highest PF of 7.85-fold and 8.41-fold, with the yield of 54.35% and 64.18% was obtained for SE and ASE, respectively. Based on SDS-PAGE, higher purity was obtained after combined partitioning systems. Protease in SE and ASE as well as their BE fractions were stable at −20 °C throughout 14 days of storage. The instability of protease was more pronounced in higher storage temperatures.

## Author contributions

Sakonwat Kuepethkaew: investigation, methodology, writing – original draft. Sappasith Klomklao: conceptualization, investigation, writing – review & editing. Yi Zhang: writing – review & editing. Benjamin K. Simpson: conceptualization, supervision.

## Conflicts of interest

The authors declare that they have no known competing financial interests or personal relationships that could have appeared to influence the work reported in this paper.

## Supplementary Material
